# An mHealth-Based Health Management Information System Among Health Workers in Volta and Eastern Regions of Ghana: Pre-Post Comparison Analysis

**DOI:** 10.2196/29431

**Published:** 2022-08-31

**Authors:** Young-ji Lee, Seohyun Lee, SeYeon Kim, Wonil Choi, Yoojin Jeong, Nina Jin Joo Rhim, Ilwon Seo, Sun-Young Kim

**Affiliations:** 1 Department of Public Health Sciences, Graduate School of Public Health Seoul National University Gwanak Campus Seoul Republic of Korea; 2 Department of Global Public Administration Yonsei University Mirae Campus Wonju Republic of Korea; 3 Good Neighbors International Seoul Republic of Korea; 4 Good Neighbors Ghana Accra Ghana; 5 Institute of Health and Environment Seoul National University Gwanak Campus Seoul Republic of Korea

**Keywords:** mobile health, mHealth, e-Tracker, health information system, HIS, health information management system, HIMS, District Health Information Management System, DHIMS, maternal and child health, MCH, electronic health record, EHR, health workers

## Abstract

**Background:**

Despite the increasing attention to electronic health management information systems (HMISs) in global health, most African countries still depend on inefficient paper-based systems. Good Neighbors International and Evaluate 4 Health have recently supported the Ghana Health Service on the rollout of a mobile health–based HMIS called the *e-Tracker* system in 2 regions in Ghana. The e-Tracker is an Android-based tracker capture app that electronically manages maternal and child health (MCH) data. The Ghana Health Service has implemented this new system in Community Health Planning and Services in the 2 regions (Volta and Eastern).

**Objective:**

This study aims to evaluate changes in health workers’ capacity and behavior after using the e-Tracker to deliver MCH services. Specifically, the study assesses the changes in knowledge, attitude, and practice (KAP) of the health workers toward the e-Tracker system by comparing the pre- and postsurvey results.

**Methods:**

The KAP of frontline health workers was measured through self-administered surveys before and after using the e-Tracker system to assess their capacity and behavioral change toward the system. A total of 1124 health workers from the Volta and Eastern regions responded to the pre-post surveys. This study conducted the McNemar chi-square test and Wilcoxon signed-rank test for a pre-post comparison analysis. In addition, random-effects ordered logistic regression analysis and random-effects panel analysis were conducted to identify factors associated with KAP level.

**Results:**

The pre-post comparison analysis showed significant improvement in health workers’ capacity, with higher knowledge and practice levels after using the e-Tracker system. As for *knowledge*, there was a 9.9%-point increase (from 559/1109, 50.41% to 669/1109, 60.32%) in the proportion of the respondents who were able to generate basic statistics on the number of children born in a random month within 30 minutes. In the *practice* section, the percentage of respondents who had *scheduled client* *encounters* increased from 91.41% (968/1059) to 97.83% (1036/1059). By contrast, responses to the *attitude* (acceptability) became less favorable after experiencing the actual system. For instance, 48.53% (544/1121) initially expressed their preferences for an electronic system; however, the proportion decreased to 33.45% (375/1121) after the intervention. Random-effects ordered logistic regression showed that *days of overwork* were significantly associated with health workers’ attitudes toward the e-Tracker system.

**Conclusions:**

This study provides empirical evidence that the e-Tracker system is conducive to enhancing capacity in MCH data management for providing necessary MCH services. However, the change in attitude implies that the users appear to feel less comfortable using the new system. As Ghana plans to scale up the electronic HMIS system using the e-Tracker to the national level, strategies to enhance health workers’ attitudes are necessary to sustain this new system.

## Introduction

### Background

A health management information system (HMIS) is a critical component of the health system. According to the World Health Organization, a well-functioning HMIS should “ensure the production, analysis, dissemination, and use of reliable and timely information on health determinants, health systems performance, and health status” [1]. In other words, the key functions of HMIS include the generation, compilation, analysis, synthesis, communication, and use of health information [2]. Among these functions of HMIS, generating health data is particularly crucial as it adds value by providing insights into clinical decision-making and policy implications.

The systematic generation and management of health data in low- and middle-income countries (LMICs) have specific challenges in multiple health focus areas. For example, even the most essential vital statistics such as birth records or maternal and child health (MCH) service provision statistics have not been tracked systematically in many LMICs. In recent years, at least 15,000 newborns died annually, without official records [3]. Similarly, gaps exist between actual service provision and reported data, which makes it difficult for local governments to identify the unmet needs of health services [4]. Health data management is more challenging in resource-constrained settings as the health records are stored in paper-based charts rather than collected electronically. Health workers in such settings generate basic statistics or aggregate the data from paper-based health records and submit the data in person by visiting upper-level facilities such as district or provincial health offices. This manual process is time consuming and often leads to poor data quality [5-8]. In this context, an electronic HMIS has been recognized as an effective and efficient way of addressing this challenge and bridging the quality gap between health care service provision and data management [5,9,10]. Some African countries have recently attempted to implement mobile health (mHealth)–based HMIS as it can be operated using relatively simple software at a lower cost [11,12]. The use of mobile phones or tablet computers for operating HMIS can also address logistic problems, including limited access to fixed broadband internet [13,14], lack of electricity supply [14-17], and financial and human resource deficits in low-resource settings [9,10,18-22].

Ghana is an LMIC that has adopted an mHealth-based HMIS by implementing the MCH data capture app on a tablet computer. Originally, the HMIS in Ghana was initiated as a purely paper-based system in which all stages of data management, from data collection to storage, were performed manually. Once computers became available, the process started transitioning from paper-based to electronic systems at the district level. In 2012, Ghana Health Service (GHS), an implementing agency under the Ministry of Health, implemented the official health service data management software platform, District Health Information Management System, which enabled district health officers to manage health data electronically. However, lower-level health facilities still maintained a paper-based HMIS, which is highly error prone [14,15]. This transition in Ghana was partial as it did not include peripheral community-level health facilities [4]. Community health facilities in Ghana are called Community-based Health Planning and Services compounds and belong to the lowest level of the public health structure in Ghana [23].

In response to the growing demand for an efficient data management system, the GHS implemented the e-Tracker system in 2015, applying it first to family planning and MCH services at the community level for effective and efficient data management of the services [14,24]. The e-Tracker is an Android version of the individual client-based module in the District Health Information System 2. Developed by Oslo University in 2005, this open-source software platform enables the reporting, analysis, and sharing of data for the public health sector. The system is operated on tablet computers to resolve common obstacles such as limited electricity, internet access, lack of financing, and limited human resources by allowing offline data collection and management via portable devices [8,10,14]. GHS is taking the lead to increase health workers’ capacity not only for data recordings but also for managing tasks such as *tracking clients who drop out of care*, *scheduling*, *monitoring health services*, and *generating reports* [14,16]. Throughout these transitions, the goal of the HMIS in Ghana has been to support transparent decision-making for nationwide health sector programs [4,21].

For a successful transition from a paper-based health record to an electronic HMIS, the willingness of end users to change their workflow is essential for the sustained use of a new system. In this light, ensuring health workers’ acceptability and positive perceptions of the change in practice is one of the key facilitating factors in implementing the e-Tracker, as health workers in community health facilities are frontline workers responsible for managing health data [9,17]. Thus, health workers’ acceptability of this new system is considered a prerequisite for the successful implementation of an mHealth-based HMIS [10]. The study by Zargara et al [14] reported that a new system’s realignment of work practices is a determinant of MCH service provision quality. The study also reported that the key challenges in transitioning from paper-based to electronic health records were “an increase in workload occurred by double work” and “low computer literacy” [4,9]. A working paper published by the US Agency for International Development and Measure Evaluation showed mixed results in that the health workers from the 4 districts in Ghana’s Central Region did not use the full functionality of the new mHealth-based HMIS, such as data analysis. However, most of them were satisfied with the advanced technology for managing health data [24].

### Objective

To further investigate the frontline health workers’ capacity, perceptions, and practice toward the e-Tracker, this study conducted a pre-post survey to measure knowledge, attitude, and practice (KAP) among the health workers at Community-based Health Planning and Services compounds in the Volta and Eastern regions of Ghana where the e-Tracker was gradually rolled out to all districts within the region. The empirical findings of this study are expected to provide grounds and political implications for the national scale-up of the e-Tracker system.

## Methods

### Study Sample

This study used a quasi-experimental pre- and postanalysis design. The KAP on MCH data management using the e-Tracker was investigated through paper-based pre-post surveys. The study adopted a purposive sampling method, recruiting respondents during the e-Tracker system training sessions in the Volta (recently renamed the Oti and Volta regions) and Eastern regions in Ghana. Although there were no specific inclusion or exclusion criteria for survey participants, the respondents were presumed to possess qualifications to fulfill the research purpose as the eligible participants of the training session were frontline health workers who were in charge of providing health services and managing patient data.

For the presurvey, respondents were recruited during the initial training session of the e-Tracker system, where they were introduced to the system. The postsurvey was conducted during the refresher training after 3 to 10 months of e-Tracker use. A total of 2396 health workers participated in the presurvey; however, only 46.9% (1124/2396) of respondents who had participated in the initial training (ie, the presurvey) were able to rejoin the refresher training (ie, the postsurvey) as the GHS arranged to place a portion of the initial participants with newly employed health workers who had not received training opportunities. As a result, approximately half of the respondents from the presurvey were replaced with newly participating health workers, shrinking the study sample size (respondents who participated in both pre- and postsurveys) to 1124. The final set of respondents comprised different types of community health workers (community health nurses [CHNs] or community health officers [CHOs], midwives, enrolled nurses, and field technicians) working in the Volta and Eastern regions (Multimedia Appendix 1).

### Data Collection and the Questionnaire

The survey was conducted between October 2018 and November 2019. It was designed as a paper-based, self-administered questionnaire collected by staff from Good Neighbors International, the implementing partner of the e-Tracker training program. Responses were entered manually into a Microsoft Excel spreadsheet by the research team. The questionnaire comprised 43 multiple-choice and yes or no questions covering the content domains of demographics and KAP (Multimedia Appendix 2).

First, the *knowledge* section of the questionnaire asked respondents whether they could retrieve specific information on MCH statistics within 30 minutes. The 10 tasks listed in the questionnaire were designed based on observations during the field visit. The questions asked about the respondents’ perceived capacity to generate basic statistics (such as the number of children born, stillbirths, and women who came for antenatal care visits in a specific month in the catchment area). The first half of the questions were intended to ask whether aggregate data could be generated for a randomly selected month. The remaining 5 questions asked whether health workers could retrieve aggregate data for the month when the survey was conducted. Second, the section for *attitude* comprised 8 questions with a 5-point Likert scale to identify the level of acceptability of using an electronic device for managing MCH records. The questions asked about the respondents’ willingness, perception, and preference for using an electronic device for MCH data management. Third, the *practice* section comprised questions on the practice of 8 specific tasks related to MCH data management and the perceived difficulty in performing those tasks. In addition, the use of a tablet computer for MCH data management and the frequency of electronic devices used for MCH data management were asked. As the data on tablet computer use were systematically inaccessible, self-reported responses were used to assess the practice.

### Statistical Analysis

Data from the pre- and postsurveys coded in the spreadsheets were imported into STATA (version 14; StataCorp LLC). Unique identifications were randomly generated for each participant, which allowed each participant’s pre- and postsurvey variables to be reliably matched. McNemar chi-square and Wilcoxon signed-rank tests were used for pre-post comparison analyses. In addition, to investigate the factors associated with each KAP component, random-effects ordered logistic regression and random-effects panel analysis were conducted. For the dependent variable, a Cronbach α test was performed for each KAP to test internal consistency for aggregating different responses to a single score. The duration of the intervention (ie, use of the tablet-based e-Tracker system in managing MCH data) was selected as the explanatory variable, and the control variables were categorized into enabling environmental, demographic, and working condition factors. The explanatory variable, represented by the “number of days of using the e-Tracker system for MCH data management,” varied as the time points for the presurvey (the initial training workshop) and the postsurvey (refresher training) were different across the districts covered. The variable *days of overwork* was included only in the regression model, as introducing an mHealth-based HMIS may have intensified the health workers’ workload, increasing resistance toward the emergent system (Table 1).

**Table 1:** Analysis framework of regression analysis.

Variables		Description
**Dependent variables**		
	Knowledge	Knowledge of MCH^a^ data management (score between 0 and 10)
	Attitude	Attitude on using an electronic device to manage MCH data (scaled between 1=most negative and 5=most positive)
	Practice	Frequency of using an electronic device to manage MCH data (scaled between 1=never and 5=every time)
**Explanatory variable**		
	Duration of e-Tracker use	Days of using the e-Tracker system via a tablet computer
**Control variables**		
	Environmental factor	Level of internet connection at the health facility
	Demographic factors	Age, sex, educational level, working experience, job position, and use of mobile phone
	Working condition	Days of overwork

^a^MCH: maternal and child health.

### Ethics Approval

This study received ethics approval from the GHS Ethics Review Committee (GHS-ERC009/09/18; Multimedia Appendix 3).

## Results

### Summary of the Respondents’ Characteristics

Table 2 presents descriptive statistics for respondents who participated in the presurvey only (group A) and those who participated in both pre- and postsurveys (group B). The 2 groups of respondents were analyzed to identify any significant differences that might have been caused because of a change in sample size. In group A, approximately 53.23% (676/1272) were from the Volta region, whereas in group B, it was 31.76% (357/1124). Approximately 83.81% (1066/1272) of respondents were female in group A, whereas it was 79.27% (891/1124) for group B. As for education, both groups showed a similar proportion for each academic level; however, group A tended to have a slightly higher educational background. Specifically, the percentages of respondents with diplomas and bachelor’s degrees were about 3% points and 2% points higher for group A, respectively. Similarly, group A respondents tended to engage in a higher job position as 28.38% (361/1272) were midwives, whereas 15.84% (178/1124) were midwives in group B. Both groups had a high rate of mobile phone use as >96% indicated the *use of their own mobile phones*. As for internet access, approximately 11.4% (145/1272) and 11.48% (129/1124) of respondents answered that their facilities had no internet access, whereas 31.84% (405/1272) and 31.23% (351/1124) responded with an acceptable level of internet access at work sites groups A and B, respectively. In addition, only 6.21% (79/1272) and 6.14% (69/1124) in groups A and B, respectively, answered that their facilities had very reliable internet access. Regarding the average age and working experience, the average age of group A respondents was approximately 1 year higher than those in group B. The average duration of using an e-Tracker system was 187.55 (51.17) days. The differences between the 2 groups in the chi-square analysis results were statistically significant for all demographic factors. Notably, the results indicated that health workers with a relatively lower educational background and shorter work experience participated in both pre- and postsurveys by rejoining the refresher training.

**Table 2:** Sociodemographic characteristics of the respondents.

Characteristics			Group A, presurvey only (n=1272)		Group B, matched (n=1124)		*P* value
**Region, n (%)**							
	Volta	676 (53.23)		357 (31.76)		<.001^a^	
	Eastern	596 (46.86)		767 (68.24)		<.001^a^	
**Sex, n (%)**							
	Male	204 (16.04)		233 (20.73)		.004^a^	
	Female	1066 (83.81)		891 (79.27)		.004^a^	
	Missing	2 (0.16)		0 (0)		.004^a^	
**Educational level, n (%)**							
	Certificate	1051 (82.79)		993 (88.35)		<.001^a^	
	Diploma	179 (14.13)		121 (10.77)		<.001^a^	
	Bachelor’s degree	37 (2.92)		9 (0.8)		<.001^a^	
	Master’s degree	1 (0.08)		1 (0.09)		<.001^a^	
	Other	1 (0.08)		0 (0)		<.001^a^	
	Missing	4 (0.32)		0 (0)		<.001^a^	
**Job description, n (%)**							
	Community health nurse or community health officer	901 (70.83)		941 (83.72)		<.001^a^	
	Enrolled nurse	2 (0.16)		1 (0.09)		<.001^a^	
	Midwife	361 (28.38)		178 (15.84)		<.001^a^	
	Field technician	1 (0.08)		2 (0.18)		<.001^a^	
	Other	3 (0.24)		1 (0.09)		<.001^a^	
	Missing	4 (0.31)		1 (0.09)		<.001^a^	
**Use of mobile phone, n (%)**							
	Yes (use my own mobile phone)	1224 (96.23)		1091 (97.06)		.05^a^	
	Yes (share a mobile phone with family)	5 (0.39)		0 (0)		.05^a^	
	No (do not use or have a mobile phone)	2 (0.16)		1 (0.09)		.05^a^	
	Missing	41 (3.22)		32 (2.85)		.05^a^	
**Access to the internet, n (%)**							
	No internet	145 (11.4)		129 (11.48)		.17^a^	
	Very poor	99 (7.78)		102 (9.07)		.17^a^	
	Poor	139 (10.93)		113 (10.05)		.17^a^	
	Acceptable	405 (31.84)		351 (31.23)		.17^a^	
	Reliable	347 (27.28)		329 (29.27)		.17^a^	
	Very reliable	79 (6.21)		69 (6.14)		.17^a^	
	Missing	58 (4.56)		31 (2.76)		.17^a^	
Age (years), mean (SD)			32.89 (6.65)		31.45 (5.44)		<.001^b^
Duration of work as a health professional (years), mean (SD)			6.80 (5.74)		5.34 (5.02)		<.001^b^
Days of using an e-Tracker system, mean (SD)			N/A^c^		187.55 (51.17)		N/A

^a^*P* value derived from chi-square test.

^b^*P* value derived from 1-way ANOVA test.

^c^N/A: not applicable.

### Knowledge

The responses were analyzed using the McNemar chi-square test to evaluate the pre- and postlevel knowledge. As shown in Table 3, there were statistically significant improvements for all question items. For example, there was a 9.9%-point increase (from 559/1109, 50.41% to 669/1109, 60.32%) in the proportion of respondents who were able to *generate basic statistics within 30 minutes on the number of children born* for a randomly selected month. In addition, the proportion of respondents who were able to retrieve the *number of pregnant women expected to deliver and those scheduled for their second postnatal care visit* during the month of the survey increased by 8.9% points (from 369/1108, 33.3% to 468/1108, 42.24%) and 8.0% points (from 283/1109, 25.52% to 337/1109, 33.54%), respectively.

After obtaining an aggregated score for the levels of knowledge by summing up the total number of tasks that an individual respondent was capable of, a Cronbach α test was conducted to verify the reliability of the aggregated scores. The scale reliability coefficients of the pre- and postsurvey responses were α=.71 and α=.72, respectively. As the test results showed acceptable reliability, 10 self-reported responses were aggregated into a single score ranging between 0 and 10. A random-effects ordered logistic analysis showed no significant impact of intervention duration on health workers’ knowledge (odds ratio [OR] 1.00, 95% CI 0.99-1.00; Table 4). However, respondents’ sex, working years, and job positions had a statistically significant association with their level of knowledge. Participants who were female tended to have lower knowledge levels than participants who were male (OR 0.53, 95% CI 0.41-0.70). Moreover, health workers with longer working years had higher knowledge levels (OR 1.06, 95% CI 1.03-1.10), and compared with CHN or CHO, midwives appeared to have higher knowledge levels (OR 2.86, 95% CI 2.03-4.02).

**Table 3:** Result of pre-post analysis for knowledge (N=1109).

Knowledge on data management			Presurvey		Postsurvey		*P* value^a^
**Can retrieve basic statistics on the total number of following items for a random month within 30 minutes, n (%)**							
	Children born	559 (50.41)		669 (60.32)		<.001	
	Family planning counseling provided	723 (65.19)		783 (70.6)		.001	
	Stillbirths	282 (25.43)		354 (31.92)		<.001	
	Women visiting the facility for postpartum complications	222 (20.02)		272 (24.53)		.003	
	Women visiting for their first antenatal care	480 (43.28)		544 (49.05)		.001	
**Can retrieve basic statistics on the total number of following items during the month of the survey within 30 minutes, n (%)**							
	Defaulters for measles immunization^b^	601 (54.24)		663 (59.84)		.001	
	Pregnant women who are expected to deliver^b^	369 (33.30)		468 (42.24)		<.001	
	Children aged <l year	626 (56.45)		665 (59.16)		.05	
	Women scheduled for their second postnatal care visit	283 (25.52)		377 (33.54)		<.001	
	Women who are in their first trimester of pregnancy^b^	442 (39.89)		496 (44.77)		.002	

^a^*P* value derived from the McNemar chi-square test.

^b^A total of 1108 responses was matched.

**Table 4:** Result of regression analysis for knowledge.

Characteristics		Odds ratio (95% CI)	SE	*P* value
Days of using the e-Tracker system via tablet computer		1.00 (0.99-1.00)	0.00	.48
Age (years)		0.99 (0.97-1.02)	0.01	.67
Sex (reference: male)		0.53 (0.41-0.70)	0.07	<.001
**Education level (reference: certificate)**				
	Diploma	1.20 (0.87-1.65)	0.20	.27
	Bachelor’s degree	0.77 (0.26-2.27)	0.42	.63
	Master’s degree	0.02 (0.00-0.73)	0.04	.03
	Other	4.11 (0.12-141.19)	7.42	.43
Working years		1.06 (1.03-1.10)	0.02	.001
**Job position (reference: CHN^a^ or CHO^b^) **				
	Enrolled nurse	3.54 (0.71-17.64)	2.90	.12
	Midwife	2.86 (2.03-4.02)	0.50	<.001
	Field technician	0.18 (0.00-16.33)	0.42	.46
	Other	1.65 (0.13-21.62)	2.16	.70
**Use of mobile phone (reference: use own mobile phone)**				
	Share mobile phone	0.41 (0.04-4.08)	0.48	.44
	Do not use mobile phones	4.09 (0.27-61.14)	5.65	.31
**Access to the internet (reference: no internet)**				
	Very poor	1.30 (0.87-1.96)	0.27	.20
	Poor	1.40 (0.93-2.10)	0.29	.10
	Acceptable	1.18 (0.84-1.65)	0.20	.34
	Reliable	1.15 (0.81-1.63)	0.21	.43
	Very reliable	0.78 (0.48-1.28)	0.19	.33

^a^CHN: community health nurse.

^b^CHO: community health officer.

### Attitude

The Wilcoxon signed-rank test was conducted to assess the prelevel and postlevel of attitude. The initial results showed that approximately 33.99% (379/1115) were *most willing *to manage electronic MCH records (Table 5). However, the proportion decreased to 21.26% (237/1115), whereas the neutral response increased from 18.03% (201/1115) to 28.43% (317/1115). Regarding the preference for paper-based versus electronic-based management, 48.53% (544/1121) initially expressed their preferences for electronic systems; however, the proportion decreased to 33.45% (375/1121) after the intervention. In contrast, the percentage of respondents indifferent to the 2 options increased from 15.7% (176/1121) to 26.32% (295/1121). Compared with the results of the survey, general ideas on using an electronic system or device became less favorable.

The Cronbach α test was conducted to verify the reliability of the 5-point Likert scale for attitude levels. The scale reliability coefficients of the pre- and postsurvey responses were α=.80 and α=.85, respectively. Given the acceptable Cronbach α test results, each of the 8 answers scoring between 1 and 5 was aggregated and converted into one average value and then analyzed using a random-effect panel analysis. As shown in Table 6, the *duration of using the e-Tracker system* was positively associated with attitude toward electronic MCH data management but to a minor degree (coefficient 0.001; *P* value<.001). On the contrary, *days of overwork* showed a negative relationship with the attitude toward the new system. Regarding demographic factors, female health workers tended to favor the new system less. In addition, health workers with diplomas and bachelor’s degrees showed more positive attitudes than those with certificates. In contrast, workers with master’s degrees had less favorable attitudes. In terms of job positions, enrolled nurses had less favorable attitudes than CHNs and CHOs. Moreover, health workers who shared mobile phones with their families had less favorable attitudes than those with their own mobile phones, implying that the ownership of personal mobile phones may have equipped the respondents with adaptability to the tablet computer system. Access to the internet was also significantly associated with attitudes toward the new system. Health workers who worked at facilities with *very reliable* internet access had more favorable attitudes than those who did not. In summary, some demographic factors, such as the ownership of personal mobile phones and access to the internet, demonstrated a larger magnitude of effect on attitude than the duration of e-Tracker use.

**Table 5:** Result of pre-post analysis for attitude.

Attitude toward electronic data management			Presurvey, n (%)		Postsurvey, n (%)		*P* value^a^
**Willing to manage MCH^b^ records using an electronic system (n=1115)**							
	1 (least likely)	30 (2.69)		33 (2.96)		<.001	
	2	41 (3.68)		78 (7)		<.001	
	3 (neutral)	201 (18.03)		317 (28.43)		<.001	
	4	464 (41.61)		450 (40.36)		<.001	
	5 (most likely)	379 (33.99)		237 (21.26)		<.001	
**Comfortable with managing electronic MCH records (n=1117)**							
	1 (very uncomfortable)	28 (2.51)		32 (2.86)		<.001	
	2	46 (4.12)		106 (9.49)		<.001	
	3 (neutral)	275 (24.62)		383 (34.29)		<.001	
	4	497 (44.49)		435 (38.94)		<.001	
	5 (very comfortable)	271 (24.26)		161 (14.41)		<.001	
**Using an electronic device for managing MCH records is a good idea (n=1120)**							
	1 (strongly disagree)	6 (0.54)		16 (1.43)		<.001	
	2	6 (0.54)		34 (3.04)		<.001	
	3 (neutral)	145 (12.95)		254 (22.68)		<.001	
	4	398 (35.54)		424 (37.86)		<.001	
	5 (strongly agree)	565 (50.45)		392 (35)		<.001	
**Using an electronic device to enter MCH records is difficult for me (n=1116)**							
	1 (strongly disagree)	419 (37.54)		371 (33.24)		.70	
	2	171 (15.23)		212 (19)		.70	
	3 (neutral)	292 (26.16)		354 (31.72)		.70	
	4	187 (16.76)		145 (12.99)		.70	
	5 (strongly agree)	47 (4.21)		34 (3.05)		.70	
**I prefer using an electronic device to manage MCH records than writing them on paper (n=1121)**							
	1 (strongly disagree)	25 (2.23)		41 (3.66)		<.001	
	2	25 (2.23)		59 (5.26)		<.001	
	3 (neutral)	176 (15.7)		295 (26.32)		<.001	
	4	351 (31.31)		351 (31.31)		<.001	
	5 (strongly agree)	544 (48.53)		375 (33.45)		<.001	
**Using an electronic device to enter MCH records is more convenient than writing on paper (n=1120)**							
	1 (strongly disagree)	13 (1.16)		35 (3.13)		<.001	
	2	25 (2.23)		57 (5.09)		<.001	
	3 (neutral)	183 (16.34)		285 (25.45)		<.001	
	4	371 (33.13)		369 (32.95)		<.001	
	5 (strongly agree)	528 (47.14)		374 (33.39)		<.001	
**Using an electronic device to enter MCH records is more accurate than writing on paper (n=1120)**							
	1 (strongly disagree)	19 (1.70)		39 (3.48)		<.001	
	2	22 (1.96)		61 (5.45)		<.001	
	3 (neutral)	185 (16.52)		307 (27.41)		<.001	
	4	404 (36.07)		355 (31.7)		<.001	
	5 (strongly agree)	490 (43.75)		358 (31.96)		<.001	
**Using an electronic device to enter MCH records is more effective than writing on paper (n=1117)**							
	1 (strongly disagree)	17 (1.52)		33 (2.95)		<.001	
	2	14 (1.25)		60 (5.37)		<.001	
	3 (neutral)	169 (15.13)		295 (26.41)		<.001	
	4	415 (37.15)		384 (34.38)		<.001	
	5 (strongly agree)	502 (44.94)		345 (30.89)		<.001	

^a^*P* value derived from Wilcoxon signed-rank test.

^b^MCH: maternal and child health.

**Table 6:** Result of regression analysis for attitude.

Characteristics			Coefficient		SE		*P* value
Days of using the e-Tracker system via a tablet computer			0.001		0.00		<.001
Days of overwork			−0.01		0.00		.002
Age (years)			0.00		0.00		.58
Sex (reference: male)			−0.29		0.04		<.001
**Education level (reference: certificate)**							
	Diploma	0.10		0.05		.04	
	Bachelor’s degree	0.21		0.08		.01	
	Master’s degree	−0.19		0.05		<.001	
	Other	−0.34		0.07		<.001	
Working years			0.00		0.01		.49
**Job position (reference: CHN^a^ or CHO^b^)**							
	Enrolled nurse	−0.30		0.13		.02	
	Midwife	0.04		0.06		.45	
	Field technician	−0.28		0.22		.21	
	Other	−0.06		0.38		.88	
**Use of mobile phone (reference: use own mobile phone)**							
	Share mobile phone	−0.61		0.09		<.001	
	Do not use mobile phones	0.25		0.22		.25	
**Access to the internet (reference: no internet)**							
	Very poor	−0.09		0.07		.20	
	Poor	−0.07		0.06		.28	
	Acceptable	0.02		0.05		.70	
	Reliable	0.12		0.06		.03	
	Very reliable	0.35		0.07		<.001	

^a^CHN: community health nurse.

^b^CHO: community health officer.

### Practice

The McNemar chi-square test was conducted for self-reported use of tablet computers for MCH data management and for 8 specific tasks related to MCH data management, such as recording client demographic data or scheduling appointments (Table 7). In addition, the Wilcoxon signed-rank test was performed to assess changes in perceived difficulty in conducting each task following the adoption of the e-Tracker. As expected, the analysis showed that the use of tablet computers for MCH data management increased from 5% (56/1121) to 81.71% (916/1121). As for the frequency of electronic device use for MCH data management, most respondents (817/1119, 73.01%) answered that they had *never* used an electronic device during the presurvey; however, 26.99% (302/1119) responded that they use it *every time*, 36.73% (411/1119) for *most of the time*, 29.49% (330/1119) for *sometimes*, and 3.75% (42/1119) for *never* after the intervention (ie, during the postsurvey).

In the case of actual practice on 8 specific tasks related to MCH data management, the percentage of respondents who performed 8 tasks showed statistically significant changes after the adoption of the e-Tracker. For example, the percentage of respondents who had *scheduled client encounters* increased from 91.41% (968/1059) to 97.83% (1036/1059). In addition, the percentage of respondents who had *collected individual data into aggregates for the District Health Information Management System 2* increased from 66.04% (702/1063) to 89.93% (956/1063). When asked if they *have ever used statistical data for making a request to the District Health Office*, the percentage of respondents who answered *yes* increased from 52.28% (591/1106) to 70.02% (787/1106). However, no statistically significant changes were found for the percentages of respondents who *produce reports on MCH*, *following up health care defaulters*, and *generate basic statistics other than monthly reports on MCH*. On the one hand, the percentages of respondents who *produce reports on MCH* or *following up health care defaulters* were >97% for both pre- and postsurveys, indicating that the tasks have generally been manageable for the health care workers regardless of the e-Tracker adoption. In contrast, the percentages of respondents who had *generated basic statistics other than monthly reports on MCH* remained at approximately 78.62% (846/1076) and 77.97% (839/1076) throughout the pre- and postsurveys, respectively. This result may imply the limited use of the data aggregation functionality of the e-Tracker.

In terms of perceived difficulty for the 8 tasks, a statistically significant improvement was observed for all 8 tasks after the implementation of the e-Tracker system. For instance, 27.94% (292/1069) responded that *following up with health care defaulters* was *very difficult* before the intervention. However, after using the e-Tracker system, only 6.89% (72/1069) answered that the task was *very difficult*. Moreover, those who found the task *very easy* increased from 7.56% (79/1069) to 15.31% (160/1069).

Unlike the *knowledge* and *attitude* sections, responses from the *practice* section failed to fulfill the acceptable standard through the Cronbach α test. Thus, the practice level for regression analysis was defined as a 5-point Likert scale of the frequency of electronic device use for MCH data management, which was analyzed with random-effects ordered logistic analysis (Table 8). The results showed that health workers with diplomas (OR 1.31, 95% CI 1.02-1.67) had higher practice levels than workers with a certificate educational level. Moreover, respondents with more work experience (OR 1.06, 95% CI 1.03-1.09) tended to show higher practice levels. In the case of environmental factors, internet accessibility was associated with practice level; that is, poor (OR 1.37, 95% CI 0.97-1.93), acceptable (OR 1.61, 95% CI 1.22-2.14), and reliable (OR 1.31, 95% CI 0.98-1.76) internet access showed higher odds than no internet access.

**Table 7:** Result of pre-post analysis for practice.

Practice on MCH^a^ data management						Presurvey, n (%)			Postsurvey, n (%)			*P* value
Use a tablet computer for MCH data management (n=1121)						56 (5)			916 (81.71)			<.001^b^
**Frequency of electronic device use for MCH data management (n=1119)**												
	Every time				15 (1.34)			302 (26.99)			<.001^c^	
	Most of the time				49 (4.38)			411 (36.73)			<.001^c^	
	Sometimes				163 (14.57)			330 (29.49)			<.001^c^	
	Rarely				75 (6.7)			34 (3.04)			<.001^c^	
	Never				817 (73.01)			42 (3.75)			<.001^c^	
**The number of respondents who perform the following tasks and the perceived task difficulty**												
	**Recording client demographic data (n=1080)**				1028 (95.19)			1062 (98.33)			<.001^b^	
		**Perceived task difficulty**										
			1 (very difficult)	137 (13.33)			44 (4.14)			<.001^c^		
			2	179 (17.41)			91 (8.57)			<.001^c^		
			3	401 (39.01)			395 (37.19)			<.001^c^		
			4	222 (21.6)			321 (30.23)			<.001^c^		
			5 (very easy)	103 (10.02)			191 (71.98)			<.001^c^		
	**Scheduling client encounters (n=1059)**				968 (91.41)			1036 (97.83)			<.001^b^	
		**Perceived task difficulty**										
			1 (very difficult)	72 (7.44)			25 (2.41)			<.001^c^		
			2	153 (15.81)			77 (7.43)			<.001^c^		
			3	365 (37.71)			349 (33.69)			<.001^c^		
			4	275 (28.41)			351 (33.88)			<.001^c^		
			5 (very easy)	122 (12.6)			185 (17.86)			<.001^c^		
	**Tracking client progress over time (n=1064)**				992 (93.23)			1027 (96.52)			<.001^b^	
		**Perceived task difficulty**										
			1 (very difficult)	204 (20.56)			68 (6.62)			<.001^c^		
			2	211 (21.27)			124 (12.07)			<.001^c^		
			3	315 (31.75)			372 (36.22)			<.001^c^		
			4	215 (21.67)			319 (31.06)			<.001^c^		
			5 (very easy)	54 (5.44)			116 (11.3)			<.001^c^		
	**Following up health care defaulters (n=1069)**				1045 (97.75)			1045 (97.75)			.88^b^	
		**Perceived task difficulty**										
			1 (very difficult)	292 (27.94)			72 (6.89)			<.001^c^		
			2	264 (25.26)			132 (12.63)			<.001^c^		
			3	275 (26.32)			349 (33.4)			<.001^c^		
			4	154 (14.74)			351 (33.59)			<.001^c^		
			5 (very easy)	79 (7.56)			160 (15.31)			<.001^c^		
	**Collecting individual data into aggregates for the District Health Information Management System 2 (n=1063)**				702 (66.04)			956 (89.93)			<.001^b^	
		**Perceived task difficulty**										
			1 (very difficult)	152 (15.70)			45 (4.34)			<.001^c^		
			2	161 (16.63)			104 (10.04)			<.001^c^		
			3	213 (22)			277 (26.74)			<.001^c^		
			4	120 (12.4)			195 (18.82)			<.001^c^		
			5 (very easy)	55 (5.68)			80 (7.72)			<.001^c^		
	**Producing reports on MCH (n=1088)**				1061 (97.52)			1066 (97.98)			.30^b^	
		**Perceived task difficulty**										
			1 (very difficult)	129 (13.33)			68 (6.56)			<.001^c^		
			2	215 (22.21)			98 (9.46)			<.001^c^		
			3	375 (38.74)			403 (38.9)			<.001^c^		
			4	241 (24.9)			397 (38.32)			<.001^c^		
			5 (very easy)	103 (10.64)			135 (13.03)			<.001^c^		
	**Generating basic statistics other than monthly reports on MCH (n=1076)**				846 (78.62)			839 (77.97)			.70^b^	
		**Perceived task difficulty**										
			1 (very difficult)	92 (9.50)			41 (3.96)			<.001^c^		
			2	153 (15.81)			63 (6.08)			<.001^c^		
			3	264 (27.27)			315 (30.41)			<.001^c^		
			4	124 (12.81)			190 (18.34)			<.001^c^		
			5 (very easy)	33 (3.41)			57 (5.5)			<.001^c^		
	Ever used statistical data for making a request to the District Health Office (n=1106)				591 (52.28)			787 (70.02)			<.001^b^	

^a^MCH: maternal and child health.

^b^*P* value derived from the McNemar chi-square test.

^c^*P* value derived from the Wilcoxon signed-rank test.

**Table 8:** Result of regression analysis for practice.

Practice		Odds ratio (95% CI)	SE	*P* value
Days of using the e-Tracker system via tablet computer		1.00 (1.001-1.004)	0.00	.002
Age (years)		0.98 (0.95-1.00)	0.01	.04
Sex (reference: male)		0.83 (0.68-1.01)	0.08	.06
**Education level^a^ (reference: certificate)**				
	Diploma	1.31 (1.02-1.67)	0.16	.03
	Bachelor’s degree	0.64 (0.27-1.51)	0.28	.31
	Master’s degree	0.98 (0.13-7.56)	1.02	.98
	Other^a^	0	0	0
Working years		1.06 (1.03-1.09)	0.01	<.001
**Working position (reference: CHN^b^ or CHO^c^)**				
	Enrolled nurse	2.59 (0.70-9.53)	1.72	.15
	Midwife	0.92 (0.71-1.18)	0.12	.50
	Field technician	0.00 (0.00-0.00)	0.00	.99
	Other	1.29 (0.16-10.18)	1.36	.81
**Use of mobile phone (reference: use own mobile phone)**				
	Share mobile phone	2.98 (0.44-20.19)	2.91	.26
	Do not use mobile phones	1.22 (0.14-10.43)	1.33	.86
**Access to the internet (reference: no internet)**				
	Very poor	1.21 (0.86-1.70)	0.21	.29
	Poor	1.37 (0.97-1.93)	0.24	.07
	Acceptable	1.61 (1.22-2.14)	0.23	.001
	Reliable	1.31 (0.98-1.76)	0.19	.07
	Very reliable	1.11 (0.605-0.74)	0.23	.52

^a^The subcategory of *Other* was removed because of a low number of observations.

^b^CHN: community health nurse.

^c^CHO: community health officer.

## Discussion

### Principal Findings

This study is the first empirical analysis to explore the change in the KAP of health workers in managing MCH data using the e-Tracker system in Ghana. The pre-post comparison analysis results showed a statistically significant improvement in health workers’ knowledge and practice levels of MCH data management. Regarding *knowledge*, the proportion of respondents who reported that they could *retrieve basic MCH statistics* increased after using the e-Tracker system. The changes in the practice level were notable in that there were statistically significant increases in the number of health workers engaging in 8 MCH data management tasks, such as scheduling patients’ encounters and tracking patients’ progress. Furthermore, a significant improvement was observed in the perceived difficulty of performing these 8 tasks. These results were confirmed by a previous study that reported amelioration in the quality of newborn care of health workers in Malawi after using an mHealth solution called NeoTree [3]. In the case of *attitude*, the level remained positive after using the e-Tracker, which was in line with a previous study that identified high satisfaction with e-Tracker use [24]. However, compared with the results from the presurvey, general ideas on using an electronic system or device became less favorable after experiencing the actual system. An additional regression analysis found that the duration of the intervention (days of using a tablet computer) was positively associated with attitude and practice but to a minor degree. Most importantly, the *days of overwork* showed a statistically significant correlation with attitude level, implying the negative impact of increased workload on health workers’ acceptability. This can be explained by the concurrent use of the traditional manual and the new e-Tracker system, which created extra work for health workers, affecting their attitude toward the system. A previous study also identified the realignment of work practice and increased workload because of the introduction of the new system [4]. Furthermore, an environmental factor such as access to the internet was also an essential condition as health workers who worked at facilities with relatively more reliable internet access had more favorable attitudes and higher practice levels. This was confirmed by previous studies, which ascertained limited access to fixed broadband internet [13,14] and lack of electricity supply [14-17] as obstacles to implementing an electronic HMIS.

### Limitations

Despite its contributions in providing empirical evidence on KAP for the new technology, this study has several limitations. First, the results of this study are not free of external validity issues. The participants of this study were limited to health workers at community health facilities in Ghana, and the surveys were conducted during the training sessions for the e-Tracker adoption. Furthermore, the unexpected change in participants during the refresher training reduced the sample size, as only 46.9% (1124/2396) of the presurvey respondents responded to the postsurvey. The major problem was the demographic description of the 2 groups, which showed a statistically significant difference for every demographic factor. This implied that those who participated in both presurveys and postsurveys tended to be less experienced, which could have affected the results. Second, this study failed to establish a complete study environment to compare before and after the e-Tracker system because of the concurrent use of traditional paper-based and new electronic methods during the study period. Such a dual system caused a double burden for data management tasks on health workers, which was presumed to be the cause of less favorable responses in the *attitude* section. This was supported by the regression analysis, which found that the *days of overwork* had a negative association with the overall attitude toward the electronic-based system. Finally, this study focused on quantitative analysis and did not identify the contextual factors that could be captured through in-depth interviews. Thus, further assessment is necessary to understand the complex reasons behind the reluctance or preference for the new system.

### Policy Implications

Nevertheless, our study provides insights for drawing policy recommendations to settle the mHealth-based HMIS in Ghana. The findings warrant the benefits of the e-Tracker system, an enhancement in health workers’ capacity for MCH data management, which provides justification for the scale-up of the system. To achieve a successful adaptation of the new system, it is necessary to establish national, regional, and facility-level strategies to address users’ acceptability. First, ensuring health workers’ acceptability is pivotal for the sustained use of the advanced system [9,17]. Previous studies have concluded that double work is one of the challenges of the e-Tracker [4,9,21]. Thus, GHS needs to spur the complete replacement of manual-based data management with the e-Tracker system to enhance job efficiency by reducing the double burden at the national level. Moreover, an effort to develop the infrastructure and environment of community health facilities to secure stable internet access is necessary. Second, on-site training for health workers to use the system should be arranged regularly by the District Health Offices. A previous qualitative study on health workers’ perceptions reported that workers who were more accustomed to mobile technology tended to have a positive attitude toward an mHealth system [18]. Other studies have also reported *low computer literacy* as one of the key challenges in transitioning from paper-based to electronic health records [4,9]. Thus, training health workers in data management, defined as collecting, recording, analyzing, and reporting health data, is crucial for more accurate and reliable information and sustained system use [19,25,26]. Finally, facilitative supervision and organizational management are essential to increase users’ perceived ease and realign health workers’ tasks, which are detrimental to the sustained use of the e-Tracker system [24].

### Conclusions

Strengthening the HMIS is vital for improving health outcomes, as it facilitates communication within the health system and contributes to sound and evidence-based decision-making in health policy. However, many low-income countries rely on manual-based HMIS, which has many limitations for collecting and managing health data. The introduction of the e-Tracker, an mHealth-based HMIS, is expected to be an innovative attempt to bridge the gap between existing technology and the outdated practice of paper-based health data management. Currently, there are ongoing efforts to scale up the e-Tracker system nationally in Ghana. This context warrants an increased need to evaluate the new system’s effectiveness and sustainability by exploring health workers’ capacity and behavioral changes in using the e-Tracker system. The findings of this study will contribute to the successful adoption of the e-Tracker system at the national level by providing grounds for national scale-up and schemes to enhance the sustainability of the system.

## Appendix

Multimedia Appendix 1 Number of respondents by region and district.

Multimedia Appendix 2 Evaluation of the mobile health program questionnaire.

Multimedia Appendix 3 Ethics approval.

## Abbreviations

CHN: community health nurse
CHO: community health officer
GHS: Ghana Health Service
HMIS: health management information system
KAP: knowledge, attitude, and practice
LMIC: low- and middle-income country
MCH: maternal and child health
mHealth: mobile health
OR: odds ratio
