# Supplementary material for: An mHealth-Based Health Management Information System Among Health Workers in Volta and Eastern Regions of Ghana: Pre-Post Comparison Analysis
Source: JMIR Med Inform. 2022 Aug 31;10(8):e29431. doi: 10.2196/29431 (PMC9475412; doi:10.2196/29431)
Supplement: Multimedia Appendix 1 [file medinform_v10i8e29431_app1.docx]

**[Appendix 1] Number of respondents by Region and district**

| **Volta Region- 25 District** | **No. of respondents** | **Eastern Region- 26 District** | **No. of respondents** |
| --- | --- | --- | --- |
| Adaklu District | 12 | Akuapim South District | 29 |
| Afadjato South District | 14 | Akuapim North District | 15 |
| Agotime Ziope District | 11 | Akyemansa District | 64 |
| Akatsi North District | 10 | Asuogyaman District | 22 |
| Akatsi South District | 29 | Ayensuano District | 21 |
| Biakoye District | 18 | Atiwa District* | 24(25) |
| Central Tongu District | 10 | Birim Central Municipal District | 23 |
| Ho Municipal District** | 29(30) | Birim North District | 21 |
| Ho West District | 10 | Birim South District | 42 |
| Hohoe Municipal District | 23 | Denkyembour District | 15 |
| Jasikan District | 19 | East Akim Municipal District* *** | 16(17) |
| Kadjebi District | 16 | Fanteakwa District | 16 |
| Keta Municipal District | 14 | Kwaebibirem District | 56 |
| Ketu North District* | (1) | Kwahu Afram Plains North District | 28 |
| Ketu South Municipal District | 15 | Kwahu Afram Plains South District | 31 |
| Kpando Municipal District | 10 | Kwahu East District | 14 |
| Krachi East District | 15 | Kwahu South District | 18 |
| Krachi Nchumuru District | 6 | Kwahu West Municipal District | 38 |
| Krachi West District | 11 | Lower Manya Krobo District** | 41(42) |
| Nkwanta North District | 13 | New-Juaben Municipal District | 43 |
| Nkwanta South District | 13 | Nsawam Adoagyire Municipal District | 42 |
| North Dayi District* | 17 | Suhum Municipal | 26 |
| North Tongu District** | 12 | Upper Manya Krobo District** | 24 |
| South Dayi District | 11 | Upper West Akim District | 18 |
| South Tongu District | 19 | West Akim Municipal District*** | 35 |
|  |  | Yilo Krobo Municipal ** | 45(46) |
| **Total** | **357** | **Total** | **767** |
| * Worked at Ketu North during Pre- survey  **Worked at Ho Municipality During Pre- survey | | * Worked at Atiwa during Pre- survey  ** Worked at Lower Manya Krobo, Yilo Krobo Pre survey  *** Worked at East Akim during Pre- survey | |
