# Supplementary material for: An mHealth-Based Health Management Information System Among Health Workers in Volta and Eastern Regions of Ghana: Pre-Post Comparison Analysis
Source: JMIR Med Inform. 2022 Aug 31;10(8):e29431. doi: 10.2196/29431 (PMC9475412; doi:10.2196/29431)
Supplement: Multimedia Appendix 2 [file medinform_v10i8e29431_app2.docx]

**Date:**

**Evaluation of the mHealth Program to Support**

**Improvement of Maternal and Child Health in Ghana**

**(Questionnaire for Community Health Nurse/Community Health Officer)**

**Survey Number:**

**Section 1. District/Demographic information**

**1. Which district do you work for?**

|  |
| --- |

**2. How old are you this year (2018)?**

| **( ) years old** |
| --- |

**4. What is the highest level of education you have received?**

| ① | Certificate | ④ | Master’s degree |
| --- | --- | --- | --- |
| ② | Diploma | ⑤ | Other ( ) |
| ③ | Bachelor’s degree |  |  |

**3. What is your sex?**

| ① | Male | ② | Female |
| --- | --- | --- | --- |

**5. How many years have you worked in the field of maternal and child health?**

( ) Years and ( ) Months

**6. Which of the following position do you belong to?**

| ① | CHN/CHO |
| --- | --- |
| ② | Enrolled nurse |
| ③ | Midwife |
| ④ | Field technician |
| ⑤ | Other ( ) |

**Section 2. Technology Literacy**

**7. Do you use a mobile phone?**

| ① | Yes- I use my own mobile phone. | **What type of mobile phone do you use?**  **□Smartphone**  **□Feature phone (yam phone)** | |
| --- | --- | --- | --- |
| ② | Yes- I share a mobile phone with my family. |  |  |
| ③ | No- I do not use/have a mobile phone. | |  |

**8. During the last 1 month, which of the following methods did you use most frequently to report the data collected from your health facility? (Please check all that you have used.)**

| ① | Landline telephone | |
| --- | --- | --- |
| ② | Mobile phone | **Check all that you have used for your work.**  **□SMS □Email □Phone call**  **□Whatsapp □Other ( )** |
| ③ | Tablet computer |  |
| ④ | Desktop computer |  |
| ⑤ | Reported data in a paper-based form | |

**9. Do you have access to the internet at your health facility?**

| ① | Yes | ② | No |
| --- | --- | --- | --- |

**Move to Section3 (next page)**

**10. How reliable/stable do you find the internet connection at your facility?**

| ① | Very poor, it gets disconnected very frequently |
| --- | --- |
| ② | Poor, it gets disconnected frequently |
| ③ | Acceptable, but it gets disconnected sometimes |
| ④ | Reliable, but it gets disconnected once in a while |
| ⑤ | Very reliable, it hardly gets disconnected |

**11. How would you rate the speed of the internet at your facility scaling from 1to 5?.**

**(1=Very slow, 5= Excellent)**

1 2 3 4 5

**Section 3. Knowledge on Data Management**

**12. If you have 30 minutes before reporting to the District Health Office, which of the following statistics do you think you can prepare before it? (All of the numbers ONLY refer to the health facility that you work in).**

| **Statistics** | **Answer** |
| --- | --- |
| **12-1 Total number of children born in March 2018** | **□** |
| **12-2 Total number of family planning counseling provided in March 2018** | **□** |
| **12-3 Total number of stillbirths in March 2018** | **□** |
| **12-4 Total number of women visited the facility for postpartum complications in March 2018** | **□** |
| **12-5 Total number of women visited for their first antenatal care in March 2018** | **□** |
| **12-6 Total number of defaulters for measles immunization this month** | **□** |
| **12-7 Total number of pregnant women who are expected to deliver this month** | **□** |
| **12-8 Total number of children aged less than l year-old as of this month** | **□** |
| **12-9 Total number of women scheduled for their 2nd postnatal care visit this month** | **□** |
| **12-10 Total number of women who are in their first trimester of pregnancy as of this month** | **□** |

**Please rate your answer scaling from 1 to 5.**

1. **How willing are you in managing maternal and child health (MCH) records electronically?**

**(1= Least Likely, 5=Most Likely)**

1 2 3 4 5

1. **How comfortable are you with managing electronic MCH records?** (ex: editing, saving, deleting the data)

**(1= Very uncomfortable, 5=very comfortable)**

1 2 3 4 5

| 1. **I think using an electronic device for managing MCH records is a good idea.** | \| **Strongly disagree** \|  \| **Neutral** \|  \| **Strongly agree** \| \| --- \| --- \| --- \| --- \| --- \| \| **1** \| **2** \| **3** \| **4** \| **5** \| |
| --- | --- | --- | --- | --- | --- | --- | --- | --- | --- | --- | --- |
| 1. **Using an electronic device to enter MCH records is difficult for me.** | \| **Strongly disagree** \|  \| **Neutral** \|  \| **Strongly agree** \| \| --- \| --- \| --- \| --- \| --- \| \| **1** \| **2** \| **3** \| **4** \| **5** \| |
| 1. **I prefer using electronic device to manage MCH records than writing them on paper.** | \| **Strongly disagree** \|  \| **Neutral** \|  \| **Strongly agree** \| \| --- \| --- \| --- \| --- \| --- \| \| **1** \| **2** \| **3** \| **4** \| **5** \| |
| 1. **Using an electronic device to enter MCH records is more convenient than writing on paper.** | \| **Strongly disagree** \|  \| **Neutral** \|  \| **Strongly agree** \| \| --- \| --- \| --- \| --- \| --- \| \| **1** \| **2** \| **3** \| **4** \| **5** \| |
| 1. **Using an electronic device to enter MCH records is more accurate than writing on paper.** | \| **Strongly disagree** \|  \| **Neutral** \|  \| **Strongly agree** \| \| --- \| --- \| --- \| --- \| --- \| \| **1** \| **2** \| **3** \| **4** \| **5** \| |
| 1. **Using an electronic device to enter MCH records is more effective than writing on paper.** | \| **Strongly disagree** \|  \| **Neutral** \|  \| **Strongly agree** \| \| --- \| --- \| --- \| --- \| --- \| \| **1** \| **2** \| **3** \| **4** \| **5** \| |

**Section 4. Attitude on Data Management**

**Section 5. Practice on Data Management**

**21. Have you used any of the following electronic devices for managing maternal and child health (MCH) data? (Please check all that you have used before)**

| **□** | Desktop computer |
| --- | --- |
| **□** | Tablet computer |
| **□** | Laptop |
| **□** | Mobile phone |
| **□** | Other ( ) |
| **□** | Never used any electronic device for data management |

**22. How often do you use electronic devices to manage MCH data?**

| ① | Every time |
| --- | --- |
| ② | Most of the time |
| ③ | Sometimes |
| ④ | Rarely |
| ⑤ | Never |

**23. How long does it take for you to identify the health records for a one particular pregnant woman who visited three months ago from the ANC register? Tick the box and please write the amount of time taken.**

| **□** paper-based register | ( ) minutes |
| --- | --- |
| **□** electronic record | ( ) minutes |

**24. How long does it take for you to identify the health records for a one particular child who visited three months ago from the CWC register? Tick the box and please write the amount of time taken**

| **□** paper-based register | ( ) minutes |
| --- | --- |
| **□** electronic record | ( ) minutes |

**25. Have you experienced any changes in the amount of your workload over the past 3 months,?**

| ① | Yes | **25-1 Did it increase or decrease?**  ① Increase in workload  ② decrease in workload |
| --- | --- | --- |
| ② | No |  |
| ③ | Don’t know |  |

**26. During the past month, if there was any, how many day(s) have you overworked (exceeded your working hour)?**

**( )day(s)**

**27. Have you conducted any of the following tasks mentioned below while working at CHPS? Please indicate either Yes or No if you have conducted any of the following tasks listed below.**

| **Task** | **Answer** | |
| --- | --- | --- |
| **27-1 Registration of client demographic data** | ① Yes  ② No | **27-1-1 How would you rate the difficulty of this task?**   \| **Very difficult** \|  \| **Neutral** \|  \| **Very**  **Easy** \| \| --- \| --- \| --- \| --- \| --- \| \| **1** \| **2** \| **3** \| **4** \| **5** \| |
| **27-2 Scheduling client encounters** | ① Yes  ② No | **27-2-1 How would you rate the difficulty of this task?**   \| **Very difficult** \|  \| **Neutral** \|  \| **Very**  **Easy** \| \| --- \| --- \| --- \| --- \| --- \| \| **1** \| **2** \| **3** \| **4** \| **5** \| |
| **27-3 Tracking client progress over time in accessing health care** | ① Yes  ② No | **27-3-1 How would you rate the difficulty of this task?**   \| **Very difficult** \|  \| **Neutral** \|  \| **Very**  **Easy** \| \| --- \| --- \| --- \| --- \| --- \| \| **1** \| **2** \| **3** \| **4** \| **5** \| |
| **27-4 Following up on healthcare defaulters** | ① Yes  ② No | **27-4-1 How would you rate the difficulty of this task?**   \| **Very difficult** \|  \| **Neutral** \|  \| **Very**  **Easy** \| \| --- \| --- \| --- \| --- \| --- \| \| **1** \| **2** \| **3** \| **4** \| **5** \| |
| **27-5 Collecting individual data into aggregates for the DHIMS2** | ① Yes  ② No | **27-5-1 How would you rate the difficulty of this task?**   \| **Very difficult** \|  \| **Neutral** \|  \| **Very**  **Easy** \| \| --- \| --- \| --- \| --- \| --- \| \| **1** \| **2** \| **3** \| **4** \| **5** \| |

| **27-6 Producing reports on MCH** | ① Yes  ② No | **27-6-1 How would you rate the difficulty of this task?**   \| **Very difficult** \|  \| **Neutral** \|  \| **Very**  **Easy** \| \| --- \| --- \| --- \| --- \| --- \| \| **1** \| **2** \| **3** \| **4** \| **5** \|   **27-6-2 Check all that you have done.**  **□Due list**  **□Defaulter list**  **□ANC coverage analyses**  **□PNC coverage analyses**  **□Delivery**  **□Family Planning**  **□Vaccination drop-out analyses for children**  **□Other ( )** |
| --- | --- | --- | --- | --- | --- | --- | --- | --- | --- | --- | --- | --- |

| **27-7 Generating basic statistics other than monthly report on MCH** | ① Yes  ② No | **27-7-1 How would you rate the difficulty of this task?**   \| **Very difficult** \|  \| **Neutral** \|  \| **Very**  **Easy** \| \| --- \| --- \| --- \| --- \| --- \| \| **1** \| **2** \| **3** \| **4** \| **5** \|   **27-7-2 Check all that you have done.**  **□Due list**  **□Defaulter list**  **□ANC coverage analyses**  **□PNC coverage analyses**  **□Delivery**  **□Family Planning**  **□Vaccination drop-out analyses for children**  **□Other ( )** |
| --- | --- | --- | --- | --- | --- | --- | --- | --- | --- | --- | --- | --- |

**28. Have you ever used statistical data for making a request to the District Health Office? For example, the use of vaccination coverage rate when requesting for the vaccines that are out of stock.**

| ① | Yes | If yes, what kind of statistical data did you use?  ( ) |
| --- | --- | --- |
| ② | No | End of the survey |
