# Supplementary material for: An mHealth-Based Health Management Information System Among Health Workers in Volta and Eastern Regions of Ghana: Pre-Post Comparison Analysis
Source: JMIR Med Inform. 2022 Aug 31;10(8):e29431. doi: 10.2196/29431 (PMC9475412; doi:10.2196/29431)
Supplement: Multimedia Appendix 3 [file medinform_v10i8e29431_app3.pdf]

GHANA HEALTH SERVICE ETHICS REVIEW COMMITTEE

In case of reply the number and date of this Letter should be quoted.

Research & Development Division
Ghana Health Service
P. O. Box MB 190
Accra
Tel: +233-302-681109
Fax + 233-302-685424
Email: ghserc@gmail.com

19th October, 2018

MyRef. GHS/RDD/ERC/Admin/App 18/418
Your Ref. No.

Sun -Young Kim
Graduate School of Public Health
Seoul National University
Gwanak-ro, Gwanak-gu, Seoul
Republic of Korea

The Ghana Health Service Ethics Review Committee has reviewed and given approval for the implementation of your Study Protocol.

GHS-ERC Number	**GHS-ERC009/09/18**
Project Title	Evaluation of the mHealth Program to Support Improvement of Maternal and Child in Ghana
Approval Date	19th October,2018
Expiry Date	18th October, 2019
GHS-ERC Decision	**Approved**

This approval requires the following from the Principal Investigator

- Submission of yearly progress report of the study to the Ethics Review Committee (ERC)
- Renewal of ethical approval if the study lasts for more than 12 months,
- Reporting of all serious adverse events related to this study to the ERC within three days verbally and seven days in writing.
- Submission of a final report **after completion** of the study
- Informing ERC if study cannot be implemented or is discontinued and reasons why
- Informing the ERC and your sponsor (where applicable) before any publication of the research findings.

Please note that any modification of the study without ERC approval of the amendment is invalid.

The ERC may observe or cause to be observed procedures and records of the study during and after implementation.

Kindly quote the protocol identification number in all future correspondence in relation to this approved protocol

SIGNED..

PROFESSOR MOSES AIKINS
(GHS-ERC VICE CHAIRPERSON)
